# Supplementary material for: Delays in the presentation and diagnosis of women with breast cancer in Yogyakarta, Indonesia: A retrospective observational study
Source: PLoS One. 2022 Jan 13;17(1):e0262468. doi: 10.1371/journal.pone.0262468 (PMC8757982; doi:10.1371/journal.pone.0262468)
Supplement: S1 File — (DOC) [file pone.0262468.s001.doc]

| **INTERVIEWER ID:**   |  |  |  | | --- | --- | --- | | | | **CONFIDENTIAL** | **RESPONDENT ID:**   |  |  |  |  |  |  |  |  |  |  |  | | --- | --- | --- | --- | --- | --- | --- | --- | --- | --- | --- | | |
| --- | --- | --- | --- | --- | --- | --- | --- | --- | --- | --- | --- | --- | --- | --- | --- | --- | --- | --- | --- |
| **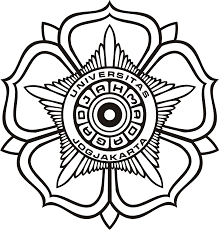** | | | | | |
| **Start of interview date (dd/mm/yy)** | | | **....../...../20 Time…………………….WIB** | | |
| **End of interview date (dd/mm/yy)** | | | **....../...../20 Time…………………….WIB** | | |
|  |  | Question Number **(cp1no)** | | | Notes **(cp1notes)** |
| CP1. | Questions with dubious answers |  | | |  |
| CP2. | Questions that require changing the unit of measurement |  | | |  |
| CP3. | Questions requiring another data source (e.g. secondary data) |  | | |  |
| CP4. | Other problems |  | | |  |
| **INTERVIEWER'S NOTE** | | | | | |
|  | | | | | |

**FACULTY OF MEDICINE, PUBLIC HEALTH AND NURSING**

**UNIVERSITAS GADJAH MADA (UGM)**

***DELAY IN DIAGNOSIS OF BREAST CANCER***

**OPENING SENTENCE:** Good [morning/day/afternoon]. My name is _. I am a research assistant at the Dr. Sardjito General Hospital located in Yogyakarta. We wish to obtain data on the experience of patients regarding diagnosis and care of breast cancer. In this survey, I will ask __ questions which will approximately 20 minutes.

|  | **Section 1: Personal Information** | |
| --- | --- | --- |
| **d1** | What is your full name? |  |
| **d2a** | Where do you live according to your KTP? | - **d2aprov**. Province……………………………………. - **d2akab.** District…………………………………. - **d2akec.** Subdistrict…………………………………. |
| **d2b** | In the last 6 months, do you still live at the same address as your KTP? | - (1) Yes -> go to question d3 - (2) No. |
| **d2c** | If different, where have you lived in the last 6 months? | - **d2cprov**. Province……………………………………. - **d2ckab.** District…………………………………. - **d2ckec.** Subdistrict…………………………………. |
| **d3** | How long have you lived in your current residence?  [Note for interviewer: If the respondent can specify the period of week, month and year with precision, then all three are inputted. If not, just fill in one according to the respondent's statement] | - **d3w**. ………………………………………. week - **d3m.** ……………….……………………… month - **d3y.** ……………….……………………… year |
| **d4** | Currently, besides you, who are the people living in your house?  [note to interviewer: For d4f1-9 option, respondent may choose more than one suitable option] | - **d4f1**. Husband - **d4f2**. Children, how many people? ……………**(d4f2o)** - **d4f3**. Parents, how many people? ……..**(d4f3o)** - **d4f4**. Older brother or sister, how many people? …………..**(d4f4o)** - **d4f5.** Younger brother or sister, how many people?…………..**(d4f5o)** - (9) Others, state ………………………………… .. (d4f9who) How many people? ………………………… .. (d4f9o) - (88) NONE - (99) NOT ANSWERING - (8) DO NOT KNOW |
| **d5** | How old are you? | ….…………………..year |
| **d6** | What is the highest level of education you have taken until you graduated?  [Note to interviewers: Respondents may only choose one option] | - (1) Never had a formal education - (2) Never completed elementary school - (3) elementary school - (4) middle school - (5) high school - (6) Diploma (D1, D2, D3) - (7) S1 - (8) S2/S3 - (9) Others, …………..**(d6lain9)** - (99) NOT ANSWERING - (8) DO NOT KNOW |
| **d7** | What was the estimate net monthly salary/income/wage of your household during the past month? | - (1) ……………………………….rupiah **(d7rp)** - (99) NOT ANSWERING - (8) DO NOT KNOW |
| **d8** | What was the estimate net monthly salary/income/wage of your household during the past 12 months? | - (1) ……………………………….rupiah **(d8rp)** - (99) NOT ANSWERING - (8) DO NOT KNOW |

**Now I am going to ask you a question about your experience in accessing or visiting breast cancer related health services.**

|  | **Part 2: Access / Visit to Health Services** | |
| --- | --- | --- |
| **a1** | On what date did you first notice a change or lump in your breast? | ….……………………………………………………………………  ….……………………………………………………………………  ….…………………………………………………………………… |
| **a3** | On what date was your first visit to a conventional health care (not alternative) facility in regards to your breast complaints? *[Note to interviewer: If the respondent can state the complete date (day, week, month and year) and precision, then all three are inputted. If not, just fill in one according to the respondent's statement]* | ….……………………………………………………………………  ….……………………………………………………………………  ….…………………………………………………………………… |
| **a4** | How many times have you visited a conventional (not alternative) health facility in regards to your breast complaints? | ….………..times |
| **a5** | What types of conventional (not alternative) health facility did you visit to get yourself checked? | ….……………………………………………………………………  ….……………………………………………………………………  ….…………………………………………………………………… |
| **a6** | What type of **medical health facility / service** do you visit for the first time to have your breast complaint examined? | ….……………………………………………………………………  ….……………………………………………………………………  ….…………………………………………………………………… |
| **a7** | How long did it take between the first time you noticed changes in your breasts and the first time you visited a medical health facility?  *[Note to interviewer: If the respondent can specify the period of day, week, month and year with precision, then all four are inputted. If not, just fill in one according to the respondent's statement]* | - **a7d.** ……………………………………….day - **a7w**. ……………………………………….week - **a7m.** ……………….………………………month - **a7y.** ……………….………………………year |
| **a8** | How long did it take you to travel one way from your place of residence to the first health facility you visited?  *[Note to interviewer: one way means only going to the facility, or going home. Not going back and forth]* | - **a8min.** ……………………………………minute - **a8h.** ……………………………………hour - **a8d.** ……………………………………day - (99) NOT ANSWERING - (8) DO NOT KNOW |
| **a9** | What is the approximate distance, in kilometers, from your place of residence to the health facility? | - (1)………………………..km **(a9km)** - (8) DO NOT KNOW - (99) NOT ANSWERING |
| **a10** | Approximately how much does it cost on a one-way trip from your place of residence to the health facility?  *[Note to interviewer: travel costs include gasoline, vehicle rental, driver rental, and / or taxi / bus / ojek costs]* | - (1)………………………..rupiah **(a10rp)** - (3) I walk to the health facility - (8) DO NOT KNOW - (99) NOT ANSWERING |
| **a11** | Did you seek alternative treatment first before getting yourself checked at a healthcare facility in regards to your breast complaints?  *[Note to interviewer: Alternative medicine means treatment outside of medical treatment, for example shaman, massage, herbal medicine, shinshe, acupuncture, spiritual approach]* | - (1) Yes - (2) No -> **go to question a14** - (8) DO NOT KNOW / NOT SURE -> **go to question a14** - (99) NO ANSWERING -> **go to question a14** |
| **a12** | What kind of alternative treatments did you seek? *[Note to interviewer: Alternative medicine means treatment outside of medical treatment, for example shaman, massage, herbal medicine, shinshe, acupuncture, spiritual approach]* | ….……………………………………………………………………  ….……………………………………………………………………  ….……………………………………………………………………  ….……………………………………………………………………  ….……………………………………………………………………  ….……………………………………………………………………  ….……………………………………………………………………  ….……………………………………………………………………  ….…………………………………………………………………… |
| **a13** | How many times did you visit the alternative treatment provider? | - (1)………………………………times **(a13freq)** - (8) DO NOT KNOW / NOT SURE |
| **a14** | Do you take herbal medicine / traditional medicine / herbal medicine / empon-empon / to treat your breast complaints? | - (1) Yes - (2) No -> **go to question a18** - (8) DO NOT KNOW / NOT SURE -> **go to question a18** - (99) NO ANSWERING -> **go to question a18** |
| **a15** | What types of herbal medicine / traditional medicine / herbal medicine / empon-empon do you consume? | ….……………………………………………………………………  ….……………………………………………………………………  ….……………………………………………………………………  ….……………………………………………………………………  ….……………………………………………………………………  ….…………………………………………………………………… |
| **a16** | How much did you take and how often did you take the herbs / traditional medicines / herbs / empon-empon? | - (1) ……………………………… kali **(a16freq)** - (8) FORGOT / DO NOT KNOW |
| **a17** | At what time of day do you take these herbs / traditional medicines / herbs / empon-empon?  *[note to interviewer: please find out whether the herbal medicine was consumed before diagnosis, or after diagnosis in conjunction with medical therapy]* | ….……………………………………………………………………  ….……………………………………………………………………  ….……………………………………………………………………  ….……………………………………………………………………  ….……………………………………………………………………  ….……………………………………………………………………  ….……………………………………………………………………  ….…………………………………………………………………… |
| **a18** | What were the reasons for delaying getting yourself checked in regards to your breast complaints?  *[note to interviewer: Delay = from the moment she realizes breast changes until she goes to a medical / conventional health facility; only asked to those who delayed diagnosis]* | ….……………………………………………………………………  ….……………………………………………………………………  ….……………………………………………………………………  ….……………………………………………………………………  ….……………………………………………………………………  ….……………………………………………………………………  ….……………………………………………………………………  ….…………………………………………………………………… |

**I will now ask about your knowledge and attitudes related to breast cancer.**

|  | **Section 3: Knowledge, Attitudes, and Actions / Practices Related to Breast Cancer** | |
| --- | --- | --- |
| **kap1** | Would you name as many early warning signs of breast cancer as you can think of.  *[Note to interviewer: Subjects are welcome to state as much as they know]* | ….……………………………………………………………………  ….……………………………………………………………………  ….……………………………………………………………………  ….……………………………………………………………………  ….……………………………………………………………………  ….……………………………………………………………………  ….……………………………………………………………………  ….…………………………………………………………………… |
| **kap2** | How often do you check your breasts (SADARI)?  *[note to interviewer: "Rarely" means less than 2x a year]* | - (1) Rarely or never - (2) At least once every 6 months - (3) At least once a month - (4) At least once a week - (99) NO ANSWERING - (8) FORGET / DO NOT KNOW |
| **kap3** | Are you confident you would notice a change in your breasts? | - (1) Not at all sure about the change - (2) A little sure and confident that there will be changes - (3) Sufficiently confident and confident that there will be changes - (4) Very confident and confident that there will be changes) |
| **kap4** | Would you name as many early breast cancer risk factors as you can think of.  *[Note to interviewer: Subjects are welcome to state as many known risk factors as possible]* | ….……………………………………………………………………  ….……………………………………………………………………  ….……………………………………………………………………  ….……………………………………………………………………  ….……………………………………………………………………  ….……………………………………………………………………  ….……………………………………………………………………  ….…………………………………………………………………… |

Now I'm going to ask you a question about the feelings you've had in the past month.

|  | **Part 4: Feelings in the Last Month** | |
| --- | --- | --- |
| **pss1** | In the last month, how often have you felt that you were unable to control the important things in your life? | - (0) Never - (1) Almost never - (2) Sometimes - (3) Quite often - (4) Very often - (99) NOT ANSWERING |
| **pss2** | In the last month, how often have you felt confident about your ability to handle your personal problems? | - (0) Never - (1) Almost never - (2) Sometimes - (3) Quite often - (4) Very often - (99) NOT ANSWERING |
| **pss3** | In the last month, how often have you felt that things were going your way? | - (0) Never - (1) Almost never - (2) Sometimes - (3) Quite often - (4) Very often - (99) NOT ANSWERING |
| **pss4** | In the last month, how often have you felt difficulties were piling up so high that you could not overcome them? | - (0) Never - (1) Almost never - (2) Sometimes - (3) Quite often - (4) Very often - (99) NOT ANSWERING |

**CLOSING SENTENCE:** Thank you for your participation. This information is very valuable for the increasing the quality of breast cancer care at the Dr. Sardjito General Hospital.
