# Supplementary material for: Delays in the presentation and diagnosis of women with breast cancer in Yogyakarta, Indonesia: A retrospective observational study
Source: PLoS One. 2022 Jan 13;17(1):e0262468. doi: 10.1371/journal.pone.0262468 (PMC8757982; doi:10.1371/journal.pone.0262468)
Supplement: S2 File — (DOC) [file pone.0262468.s002.doc]

| **ID PEWAWANCARA :**   |  |  |  | | --- | --- | --- | | | | **RAHASIA** | **ID RESPONDEN :**   |  |  |  |  |  |  |  |  |  |  | | --- | --- | --- | --- | --- | --- | --- | --- | --- | --- | | |
| --- | --- | --- | --- | --- | --- | --- | --- | --- | --- | --- | --- | --- | --- | --- | --- | --- | --- | --- |
| **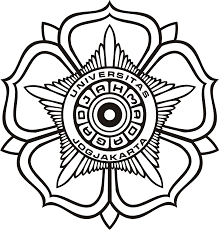** | | | | | |
| **Tanggal mulai wawancara (ddmmyy)** | | | **....../...../20 Pukul…………………….WIB** | | |
| **Tanggal selesai wawancara (ddmmyy)** | | | **....../...../20 Pukul…………………….WIB** | | |
|  |  | NOMOR PERTANYAAN **(cp1no)** | | | CATATAN **(cp1notes)** |
| CP1. | Pertanyaan dengan jawaban yang meragukan |  | | |  |
| CP2. | Pertanyaan yang membutuhkan pengubahan unit pengukuran |  | | |  |
| CP3. | Pertanyaan yang membutuhkan sumber data lain (misalnya data sekunder) |  | | |  |
| CP4. | Permasalahan lainnya |  | | |  |
| **CATATAN PEWAWANCARA** | | | | | |
|  | | | | | |

**FAKULTAS KEDOKTERAN KESEHATAN MASYARAKAT DAN KEPERAWATAN (FK-KMK)**

**UNIVERSITAS GADJAH MADA (UGM)**

**KUESIONER *DELAY IN DIAGNOSIS***

**KALIMAT PERKENALAN:** Selamat *[pagi/siang/sore]* Ibu. Nama saya ……………… Saya adalah salah satu asisten penelitian di Rumah Sakit Umum Dokter Sardjito Yogyakarta. Saat ini kami ingin melakukan pendataan terkait pengalaman mengakses pelayanan dan diagnosis kanker payudara. Dalam kegiatan ini, saya akan menanyakan …. pertanyaan dengan lama wawancara kurang lebih 20 menit.

|  | **Bagian 1: Informasi Personal** | |
| --- | --- | --- |
| **d1** | Siapa nama lengkap Anda? |  |
| **d2a** | Di mana tempat tinggal Anda sesuai dengan KTP? | - **d2aprov**. Provinsi……………………………………. - **d2akab.** Kabupaten…………………………………. - **d2akec.** Kecamatan…………………………………. |
| **d2b** | Dalam 6 bulan terakhir, apakah Anda masih tinggal di alamat yang sama sesuai dengan KTP? | - (1) Ya --> **lanjut ke pertanyaan d3** - (2) Tidak |
| **d2c** | Jika berbeda, di mana tempat tinggal Anda dalam 6 bulan terakhir? | - **d2cprov**. Provinsi……………………………………. - **d2ckab.** Kabupaten…………………………………. - **d2ckec.** Kecamatan…………………………………. |
| **d3** | Sudah berapa lama Anda tinggal di rumah Anda saat ini?  *[catatan untuk pewawancara: Jika responden bisa menyebutkan periode minggu, bulan,dan tahun secara presisi, maka ketiganya diisikan. Jika tidak, maka diisi salah satu saja sesuai keterangan responden]* | - **d3w**. ……………………………………….minggu - **d3m.** ……………….………………………bulan - **d3y.** ……………….………………………tahun |
| **d4** | Saat ini, selain Anda, siapa saja orang yang tinggal di rumah Anda?  *[catatan untuk pewawancara: Untuk opsi d4f1-9, responden boleh memilih lebih dari satu opsi yang sesuai]* | - **d4f1**. Suami - **d4f2**. Anak, berapa orang? ……………**(d4f2o)** - **d4f3**. Orang Tua, berapa orang? ……..**(d4f3o)** - **d4f4**. Kakak, berapa orang? …………..**(d4f4o)** - **d4f5.** Adik, berapa orang?…………..**(d4f5o)** - (9) Lainnya,   sebutkan…………………………………..**(d4f9who)**  berapa orang? …………………………..**(d4f9o)**   - (88) TIDAK ADA - (99) TIDAK MENJAWAB - (8) TIDAK TAHU |
| **d5** | Berapa usia Anda? | ….…………………..tahun |
| **d6** | Apa tingkat pendidikan tertinggi yang telah Anda tempuh hingga lulus?  *[catatan untuk pewawancara: Responden hanya boleh memilih satu opsi]* | - (1) Tidak pernah menempuh pendidikan formal - (2) Tidak pernah tamat SD - (3) SD - (4) SMP/SLTP umum - (5) SMU/SMA/SLTA umum/SMK - (6) Akademia (D1, D2, D3) - (7) S1 - (8) S2/S3 - (9) Lainnya, sebutkan…………..**(d6lain9)** - (99) TIDAK MENJAWAB - (8) TIDAK TAHU |
| **d7** | Berapa kira-kira gaji/upah/penghasilan bulanan bersih rumah tangga Anda selama sebulan yang lalu? | - (1) ……………………………….rupiah **(d7rp)** - (99) TIDAK MENJAWAB - (8) TIDAK TAHU |
| **d8** | Berapa kira-kira gaji/upah/penghasilan bulanan bersih rumah tangga Anda selama 12 bulan terakhir? | - (1) ……………………………….rupiah **(d8rp)** - (99) TIDAK MENJAWAB - (8) TIDAK TAHU |

**Sekarang saya akan menanyakan pertanyaan tentang pengalaman Anda dalam mengakses atau mengunjungi pelayanan kesehatan terkait kanker payudara.**

|  | **Bagian 2: Akses/Kunjungan ke Pelayanan Kesehatan** | |
| --- | --- | --- |
| **a1** | Tanggal berapa Anda pertama kali menyadari adanya perubahan atau benjolan pada payudara Anda? | ….……………………………………………………………………  ….……………………………………………………………………  ….…………………………………………………………………… |
| **a3** | Tanggal berapa Anda pertama kali mengunjungi fasilitas kesehatan (bukan pengobatan alternatif) untuk memeriksakan perubahan yang Anda perhatikan atau rasakan pada payudara Anda?  *[catatan untuk pewawancara: Jika responden bisa menyebutkan tanggal secara lengkap (hari, minggu, bulan,dan tahun) dan presisi, maka ketiganya diisikan. Jika tidak, maka diisi salah satu saja sesuai keterangan responden]* | ….……………………………………………………………………  ….……………………………………………………………………  ….…………………………………………………………………… |
| **a4** | Berapa kali Anda mengunjungi fasilitas kesehatan (bukan pengobatan alternatif) terkait dengan keluhan payudara Anda? | ….………..kali |
| **a5** | Apa saja jenis fasilitas kesehatan (bukan pengobatan alternatif) yang Anda kunjungi untuk memeriksakan diri? | ….……………………………………………………………………  ….……………………………………………………………………  ….…………………………………………………………………… |
| **a6** | Apa jenis fasilitas/layanan kesehatan medis **yang pertama kali Anda kunjungi** untuk memeriksakan keluhan payudara Anda? | ….……………………………………………………………………  ….……………………………………………………………………  ….…………………………………………………………………… |
| **a7** | Berapa jarak waktu antara pertama kali Anda menyadari perubahan payudara Anda sampai dengan pertama kali mengunjungi fasilitas kesehatan medis?  *[catatan untuk pewawancara: Jika responden bisa menyebutkan periode hari, minggu, bulan,dan tahun secara presisi, maka ketiganya diisikan. Jika tidak, maka diisi salah satu saja sesuai keterangan responden]* | - **a7d.** ……………………………………….hari - **a7w**. ……………………………………….minggu - **a7m.** ……………….………………………bulan - **a7y.** ……………….………………………tahun |
| **a8** | Berapa lama waktu yang Anda butuhkan untuk perjalanan **satu kali jalan** dari rumah Anda sampai ke fasilitas kesehatan **yang pertama Anda kunjungi** tersebut?  *[catatan untuk pewawancara: satu kali jalan artinya hanya saat pergi saja, atau pulang saja. Bukan pulang-pergi]* | - **a8min.** ……………………………………menit - **a8h.** ……………………………………menit - **a8d.** ……………………………………menit - (99) TIDAK MENJAWAB - (8) TIDAK TAHU |
| **a9** | Kira-kira berapa kilometer jarak dari rumah Anda sampai ke fasilitas kesehatan tersebut? | - (1)………………………..km **(a9km)** - (8) TIDAK TAHU - (99) TIDAK MENJAWAB |
| **a10** | Kira-kira berapa ongkos perjalanan yang Anda keluarkan untuk perjalanan **satu kali jalan** dari rumah Anda ke fasilitas kesehatan tersebut?  *[catatan untuk pewawancara: ongkos perjalanan meliputi bensin, sewa kendaraan, sewa supir, dan/atau biaya taksi/bus/ojek]* | - (1)………………………..rupiah **(a10rp)** - (3) Saya jalan kaki - (8) TIDAK TAHU - (99) TIDAK MENJAWAB |
| **a11** | Apakah anda memeriksakan keluhan payudara Anda ke pengobatan alternatif sebelum ke fasilitas kesehatan?  *[catatan untuk pewawancara: Pengobatan alternatif artinya pengobatan di luar pengobatan medis, contohnya dukun, pijat, jamu, shinshe, akupuntur, pendekatan kerohanian]* | - (1)Ya - (2) Tidak **--> lanjut ke pertanyaan a14** - (8) TIDAK TAHU/TIDAK YAKIN **--> lanjut ke pertanyaan a14** - (99) TIDAK MENJAWAB **--> lanjut ke pertanyaan a14** |
| **a12** | Jenis pengobatan alternatif apa yang Anda kunjungi?  *[catatan untuk pewawancara: Pengobatan alternatif artinya pengobatan di luar pengobatan medis, contohnya dukun, pijat, jamu, shinshe, akupuntur, pendekatan kerohanian]* | ….……………………………………………………………………  ….……………………………………………………………………  ….……………………………………………………………………  ….……………………………………………………………………  ….……………………………………………………………………  ….……………………………………………………………………  ….……………………………………………………………………  ….……………………………………………………………………  ….…………………………………………………………………… |
| **a13** | Berapa kali Anda telah melakukan kunjungan ke layanan pengobatan alternatif tersebut? | - (1)………………………………kali **(a13freq)** - (8) LUPA/TIDAK TAHU |
| **a14** | Apakah anda mengonsumsi obat herbal/obat tradisional/jamu/empon-empon/ untuk mengobati keluhan payudara Anda? | - (1)Ya - (2) Tidak **--> lanjut ke pertanyaan a18** - (8) TIDAK TAHU/TIDAK YAKIN **--> lanjut ke pertanyaan a18** - (99) TIDAK MENJAWAB **--> lanjut ke pertanyaan a18** |
| **a15** | Jenis obat herbal/obat tradisional/jamu/empon-empon apa yang Anda konsumsi? | ….……………………………………………………………………  ….……………………………………………………………………  ….……………………………………………………………………  ….……………………………………………………………………  ….……………………………………………………………………  ….…………………………………………………………………… |
| **a16** | Berapa dosis dan frekuensi Anda mengonsumsi obat herbal/obat tradisional/jamu/empon-empon tersebut? | - (1)………………………………kali **(a16freq)** - (8) LUPA/TIDAK TAHU |
| **a17** | Pada waktu kapan saja Anda mengonsumsi obat herbal/obat tradisional/jamu/empon-empon tersebut?  *[catatan untuk pewawancara: mohon digali apakah obat herbal tersebut dikonsumsi sebelum diagnosis, atau setelah diagnosis bersamaan dengan terapi medis*] | ….……………………………………………………………………  ….……………………………………………………………………  ….……………………………………………………………………  ….……………………………………………………………………  ….……………………………………………………………………  ….……………………………………………………………………  ….……………………………………………………………………  ….…………………………………………………………………… |
| **a18** | Apa alasan anda menunda untuk memeriksakan diri terkait keluhan payudara Anda?  *[catatan untuk pewawancara: Menunda=dari saat menyadari perubahan payudara sampai dia memeriksakan diri ke fasilitas kesehatan medis/konvensional; hanya ditanyakan pada yang menunda]* | ….……………………………………………………………………  ….……………………………………………………………………  ….……………………………………………………………………  ….……………………………………………………………………  ….……………………………………………………………………  ….……………………………………………………………………  ….……………………………………………………………………  ….…………………………………………………………………… |

**Sekarang saya akan menanyakan pertanyaan tentang pengetahuan Anda tentang kanker payudara.**

|  | **Bagian 3: Pengetahuan, Sikap, dan Tindakan/Praktik Terkait Kanker Payudara** | |
| --- | --- | --- |
| **kap1** | Sebutkan tanda-tanda awal dari kanker payudara sebanyak yang Anda ketahui  *[catatan untuk pewawancara: Subjek dipersilahkan menyebutkan sebanyak-banyaknya yang diketahui]* | ….……………………………………………………………………  ….……………………………………………………………………  ….……………………………………………………………………  ….……………………………………………………………………  ….……………………………………………………………………  ….……………………………………………………………………  ….……………………………………………………………………  ….…………………………………………………………………… |
| **kap2** | Seberapa sering Anda memeriksa payudara Anda sendiri (melakukan SADARI)?  *[catatan untuk pewawancara: “Jarang” artinya adalah kurang dari 2x dalam setahun}* | - (1) Jarang atau tidak pernah - (2) Setidaknya setiap 6 bulan sekali - (3) Setidaknya setiap sebulan sekali - (4) Setidaknya seminggu sekali - (99) TIDAK MENJAWAB - (8) LUPA/TIDAK TAHU |
| **kap3** | Apabila terjadi suatu perubahan pada payudara Anda, Apakah Anda yakin bahwa Anda bisa menyadari/mengenalinya? | - (1) Tidak yakin sama sekali tentang adanya perubahan - (2) Sedikit yakin dan percaya diri akan adanya perubahan - (3) Cukup yakin dan percaya diri akan adanya perubahan - (4) Sangat yakin dan percaya diri akan adanya perubahan) |
| **kap4** | Sebutkan faktor risiko dari kanker payudara sebanyak yang Anda ketahui dan dapat Anda pikirkan.  *[catatan untuk pewawancara: Subjek dipersilahkan menyebutkan sebanyak-banyaknya faktor risiko yang diketahui]* | ….……………………………………………………………………  ….……………………………………………………………………  ….……………………………………………………………………  ….……………………………………………………………………  ….……………………………………………………………………  ….……………………………………………………………………  ….……………………………………………………………………  ….…………………………………………………………………… |

Sekarang saya akan menanyakan pertanyaan perasaan yang Anda alami dalam satu bulan terakhir.

|  | **Bagian 4: Perasaan dalam Satu Bulan Terakhir** | |
| --- | --- | --- |
| **pss1** | Dalam satu bulan terakhir, seberapa sering Anda merasa tidak dapat melakukan peranan utama Anda dalam kehidupan sehari-hari? | - (0) Tidak pernah - (1) Hampir tidak pernah - (2) Kadang-kadang - (3) Cukup sering - (4) Sangat sering - (99) TIDAK MENJAWAB |
| **pss2** | Dalam satu bulan terakhir, seberapa sering Anda merasa yakin dengan kemampuan Anda mengatasi masalah-masalah pribadi Anda? | - (0) Tidak pernah - (1) Hampir tidak pernah - (2) Kadang-kadang - (3) Cukup sering - (4) Sangat sering - (99) TIDAK MENJAWAB |
| **pss3** | Dalam satu bulan terakhir, seberapa sering Anda merasa segala sesuatu telah sesuai dengan rencana Anda? | - (0) Tidak pernah - (1) Hampir tidak pernah - (2) Kadang-kadang - (3) Cukup sering - (4) Sangat sering - (99) TIDAK MENJAWAB |
| **pss4** | Dalam satu bulan terakhir, seberapa sering Anda merasa bahwa Anda menghadapi permasalahan yang Anda tidak bisa atasi? | - (0) Tidak pernah - (1) Hampir tidak pernah - (2) Kadang-kadang - (3) Cukup sering - (4) Sangat sering - (99) TIDAK MENJAWAB |

**KALIMAT PENUTUP:** Terima kasih atas partisipasi Ibu dalam pendataan ini. Informasi yang telah Ibu berikan akan sangat berarti untuk pengembangan dan peningkatan kualitas layanan kanker payudara di Rumah Sakit Umum Dokter Sardjito.
