# Supplementary figures and images for: Delays in the presentation and diagnosis of women with breast cancer in Yogyakarta, Indonesia: A retrospective observational study
Source: PLoS One. 2022 Jan 13;17(1):e0262468. doi: 10.1371/journal.pone.0262468 (PMC8757982; doi:10.1371/journal.pone.0262468)

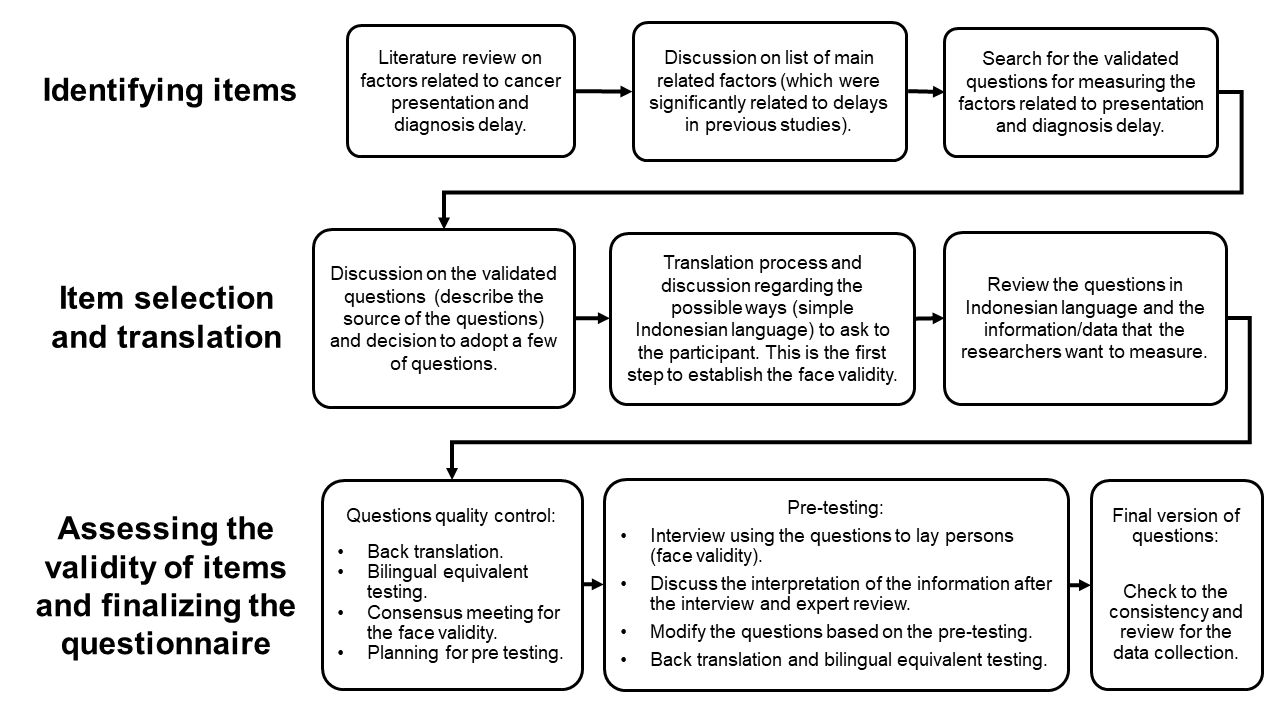

Supplement: S1 Fig — Questionnaire development started from identifying variables that will be measured and items that were obtained from existing questions related to the selected variables. It is followed by item selection and translation. The last steps included validity assessment or face validity and questionnaire finalization. (TIF) [file pone.0262468.s004.tif]
