# Supplementary material for: Delays in the presentation and diagnosis of women with breast cancer in Yogyakarta, Indonesia: A retrospective observational study
Source: PLoS One. 2022 Jan 13;17(1):e0262468. doi: 10.1371/journal.pone.0262468 (PMC8757982; doi:10.1371/journal.pone.0262468)
Supplement: S1 Table — (DOCX) [file pone.0262468.s005.docx]

**S1 Table. Proportion of presentation and diagnosis time based on stage at diagnosis.**

| **Stage** | **Time** | **<1 month**  **Freq. n (%)** | **1–<3 months**  **Freq. n (%)** | **3–<6 months Freq. n (%)** | **≥6 months**  **Freq. n (%)** |
| --- | --- | --- | --- | --- | --- |
| I–II | Presentation time | 31 (49.2) | 7 (31.8) | 5 (41.7) | 10 (18.9) |
|  | Diagnosis time | 28 (52.8) | 12 (27.9) | 5 (31.3) | 8 (21) |
| III | Presentation time | 26 (41.3) | 10 (45.5) | 6 (50) | 25 (47.2) |
|  | Diagnosis time | 16 (30.2) | 23 (53.5) | 10 (62.5) | 18 (47.4) |
| IV | Presentation time | 6 (9.5) | 5 (22.7) | 1 (8.3) | 18 (33.9) |
|  | Diagnosis time | 9 (17) | 8 (18.6) | 1 (6.2) | 12 (31.6) |
| Abbreviations: Freq =Frequency. | | | | | |
