# Supplementary material for: Delays in the presentation and diagnosis of women with breast cancer in Yogyakarta, Indonesia: A retrospective observational study
Source: PLoS One. 2022 Jan 13;17(1):e0262468. doi: 10.1371/journal.pone.0262468 (PMC8757982; doi:10.1371/journal.pone.0262468)
Supplement: S2 Table — (DOCX) [file pone.0262468.s006.docx]

**S2 Table. Effect of presentation and diagnosis delay on likelihood of lower BMI (<23) at point of diagnosis.**

| **Presence of delay** | **Likelihood of lower BMI (<23)** | | | |
| --- | --- | --- | --- | --- |
|  | **%** | **OR** | **95% CI** | **p** |
| No presentation delay | 37.7 | Ref |  |  |
| Presentation delay (≥3 months) | 55.4 | 2.08 | 1.07–4.01 | 0.030 |
| No diagnosis delay | 47.2 | Ref |  |  |
| Diagnosis delay (≥1 month) | 44.3 | 0.85 | 0.43–1.69 | 0.644 |
| Abbreviations: OR =Odds Ratio; BMI =Body Mass Index; CI =Confidence Interval. | | | | |
