# Supplementary material for: Delays in the presentation and diagnosis of women with breast cancer in Yogyakarta, Indonesia: A retrospective observational study
Source: PLoS One. 2022 Jan 13;17(1):e0262468. doi: 10.1371/journal.pone.0262468 (PMC8757982; doi:10.1371/journal.pone.0262468)
Supplement: S3 Table — (DOCX) [file pone.0262468.s007.docx]

**S3 Table. Reasons provided across all study participants for presentation delay (n =150).**

| **Reason** | **Frequency n (%)** |
| --- | --- |
| The symptoms did not bother me/caused me pain. | 43 (28.7) |
| I thought it was not serious/cancer/did not require medical attention. | 34 (22.7) |
| I was afraid of undergoing surgery. | 23 (15.3) |
| I was too busy. | 17 (11.3) |
| I was afraid of seeing a physician or going to a healthcare facility. | 9 (6) |
| I was afraid of the possible diagnosis. | 7 (4.7) |
| I sought alternative treatment first. | 6 (4) |
| I needed someone to accompany me to the healthcare facility. | 4 (2.7) |
| I was concerned about the cost. | 3 (2) |
| I was looking for a female physician. | 2 (1.3) |
| I am embarrassed if my breast has to be examined. | 2 (1.3) |
| I was afraid to going out due to COVID-19 pandemic. | 2 (1.3) |
| I needed to seek opinion from my relatives/peers. | 1 (0.7) |
| I do not think I delayed my presentation. | 38 (25.33) |
| Abbreviations: COVID-19 =Coronavirus Disease 2019. |  |
