# Supplementary material for: Delays in the presentation and diagnosis of women with breast cancer in Yogyakarta, Indonesia: A retrospective observational study
Source: PLoS One. 2022 Jan 13;17(1):e0262468. doi: 10.1371/journal.pone.0262468 (PMC8757982; doi:10.1371/journal.pone.0262468)
Supplement: Attachment — Submitted filename: Response to Reviewers.docx [file pone.0262468.s009.docx]

**Response to Reviewers**

Dear editor and reviewers,

We are thankful for the positive feedback received from Reviewers 1 and 2, and the editorial team, and for the opportunity to respond to the constructive points in our accompanying revised manuscript. Please find below our point-by-point response to reviewer feedback outlining, where relevant, the related changes we have made. We have uploaded revised versions of the manuscript as instructed, including both a clean copy and a track changes version.

***Editor comments***

1. Do follow the acceptable repositories as recommended by Plos One, not just dataset to generate figure 1, but also table 1-6. Please see <http://journals.plos.org/plosone/s/data-availability#loc-recommended-repositories>.

**Response:**

We have revised the dataset to now include deidentified data used to generate Figure 1 and Tables 1-6. We have provided derived values for date information to avoid identification of participants. Sharing of the full de-identified dataset is not possible due to restrictions imposed by the ethics committee as most of these contain patient data, albeit de-identified, and it may be possible to determine the identity of participants given the extent of sociodemographic and clinical data available for each participant.

Should there be a request for data, this can be sent to the corresponding author (email: [susanna.hutajulu@ugm.ac.id](mailto:susanna.hutajulu@ugm.ac.id)). Future researchers can contact the institutional ethics committee (email: [mhrec_fmugm@ugm.ac.id](mailto:mhrec_fmugm@ugm.ac.id)) at Universitas Gadjah Mada, Indonesia with data access queries as well.

We have included again our response to this comment in the accompanying cover letter.

2. If applicable, we recommend that you deposit your laboratory protocols in protocols.io to enhance the reproducibility of your results. Protocols.io assigns your protocol its own identifier (DOI) so that it can be cited independently in the future. For instructions see: https://journals.plos.org/plosone/s/submission-guidelines#loc-laboratory-protocols. Additionally, PLOS ONE offers an option for publishing peer-reviewed Lab Protocol articles, which describe protocols hosted on protocols.io. Read more information on sharing protocols at <https://plos.org/protocols?utm_medium=editorial-email&utm_source=authorletters&utm_campaign=protocols>.

**Response:**

Not applicable.

3. Please review your reference list to ensure that it is complete and correct. If you have cited papers that have been retracted, please include the rationale for doing so in the manuscript text, or remove these references and replace them with relevant current references. Any changes to the reference list should be mentioned in the rebuttal letter that accompanies your revised manuscript. If you need to cite a retracted article, indicate the article’s retracted status in the References list and also include a citation and full reference for the retraction notice.

**Response:**

Thank you for your comment. We have checked all references and found no article with a retraction notice.

***Reviewer 2***

**Major issues**

1. Questionnaire development (recall bias)
   The authors explained the approach to avoid recall bias as part of their response to Reviewer #1.

**Response:** Thank you for your approval.

2. The analysis (multivariable logistic regression)
   The authors made revision and updated the analyses as well as the Tables (Table 3 and 4).

**Response:**

Thank you for your approval.

3. The authors explained the reason of the analysis of molecular type of breast cancer with diagnosis delay (Tables 3 and 4). However, the literature used by the authors (Galukande 2014) is a cross-sectional study to determine the prevalence of breast cancer molecular phenotypes. In my opinion, this reference is not suitable with the purpose of the analysis made by the authors . Molecular type is an immunohistochemical classification or characterization of breast cancer for hormone receptor status or HER-2 gene overexpression and it correlates with the clinical outcome or the choice of systemic therapy (e.g. ESMO Educational sessions in Annals of Oncology vol 23 suppl 10, Sept 1,2012; National Comprehensive Cancer Network Guidelines version 4.2020; or Nature/NPJ Breast Cancer 6 March 2020). Molecular types also correlate with prognosis of breast cancer (2019 ASCO education books - Molecular testing in breast cancer by Jennifer K. Litton et al). Unfortunately, there is no relevant literature of molecular type of breast cancer in association with diagnosis delays. I am concern that this issue is the major issue of the manuscript.

**Response**:

Thank you for your thoughts on the issue. We have decided to exclude molecular subtype variables out of the multivariate model and related references until there is further evidence to determine any potential influence it may have on diagnosis delays. As a result, we have also adjusted the results in Tables 3-5 and abstract and discussion sections for the exclusion of molecular subtypes in these analyses.

**Minor issues**

The authors edited the design as suggested.

**Response:**

Thank you for your favourable opinion.
